# X-linked intellectual disability related to a novel variant of *KLHL15*

**DOI:** 10.1038/s41439-023-00248-7

**Published:** 2023-07-14

**Authors:** Jun Kido, Kimiyasu Egami, Yohei Misumi, Keishin Sugawara, Naomi Tsuchida, Naomichi Matsumoto, Mitsuharu Ueda, Kimitoshi Nakamura

**Affiliations:** 1 Department of Pediatrics, Kumamoto University Hospital, Kumamoto, Japan; 2 Department of Pediatrics, Faculty of Life Sciences, Kumamoto University, Kumamoto, Japan; 3 Egami Children Clinic, Kumamoto, Japan; 4 Department of Neurology, Kumamoto University Hospital, Kumamoto, Japan; 5 Department of Human Genetics, Yokohama City University Graduate School of Medicine, Yokohama, Japan; 6 Department of Rare Disease Genomics, Yokohama City University Hospital, Yokohama, Japan

**Keywords:** Neurodevelopmental disorders, Genetics of the nervous system

## Abstract

Kelch-like (KLHL) 15, localized on chromosome Xp22.11, was recently identified as an X-linked intellectual disability gene. Herein, we report a case of a male patient with a novel nonsense variant, c.736 C > T p.(Arg246*), in *KLHL15*, who presented with impaired intelligence, short stature, frequent hypoglycemia, and periodic fever. Patients with nonsense variants in *KLHL15* may develop intellectual disabilities, minor skeletal anomalies, and facial dysmorphisms.

The Kelch-like (*KLHL*) gene family encodes proteins that constitute a subgroup at the intersection of the BTB (BR-C, ttk, and bab) and POZ (Pox virus and zinc finger) domains and Kelch domain superfamilies^1^. *KLHL15* (MIM #300980), localized on chromosome Xp22.11, was recently identified as a gene that causes X-linked intellectual disability (XLID, MIM #300982) and functions as an adapter for cullin3 (Cul3)-based E3 ubiquitin ligases that target specific substrates of the ubiquitin‒proteasome system^2–4^. Phosphatase 2A, which is enriched in the nervous system, is targeted by the E3 ubiquitin ligase system^1^. Some reports have described the clinical phenotypes of diseases related to *KLHL15*^2,5–9^. Although patients with variants of *KLHL15* may develop intellectual disabilities, minor skeletal anomalies, and facial dysmorphism, few case reports have described the clinical manifestations of those patients. The correlation between the variant and phenotype of *KLHL15* remains uncertain. In this study, we report a case of a male patient with a novel nonsense variant, NM_030624.3:c.736 C > T p.(Arg246*), in *KLHL15*, who presented with impaired intelligence, short stature, frequent hypoglycemia, and periodic fever. In this paper, we have presented a case of a male patient with XLID and discussed the clinical manifestations of *KLHL15*-related diseases in this case and other reports.

A 6-year-old male presented with developmental delays, short stature (Fig. 1), frequent hypoglycemia, and periodic fever. The patient was born vaginally at 38 weeks and 1 day of gestation. His birth height was 47.0 cm (−0.59 SD), and his birth weight was 2498 g (−0.98 SD). He acquired a stable head at the age of 3 months, sat at 6 months, and crawled at 9 months. At 21 months, he presented with short stature (<−3.0 SD) (Fig. 1A), could only walk with support, and could not speak more than one word. Brain magnetic resonance imaging at 2 years showed no abnormal signs, including delayed myelination (Fig. 1B). The patient’s developmental quotients (DQs) on the Kyoto Scale of Psychological Development at age 5 years and 3 months corresponded with those usually found at the age of 2 years and 9 months in healthy controls (total DQ, 52; postural-motor DQ, not available; cognitive-adaptive DQ, 56; and language-social DQ, 45). From the age of five, he frequently developed ketogenic hypoglycemia (blood sugar levels < 60 mg/dL) when he could consume only a small amount of food due to a fever and/or the common cold. Moreover, his home doctor diagnosed him with periodic fever with aphthous pharyngitis and adenitis because he frequently had a lasting high fever for 1–2 weeks once or twice a month.

**Fig. 1:**
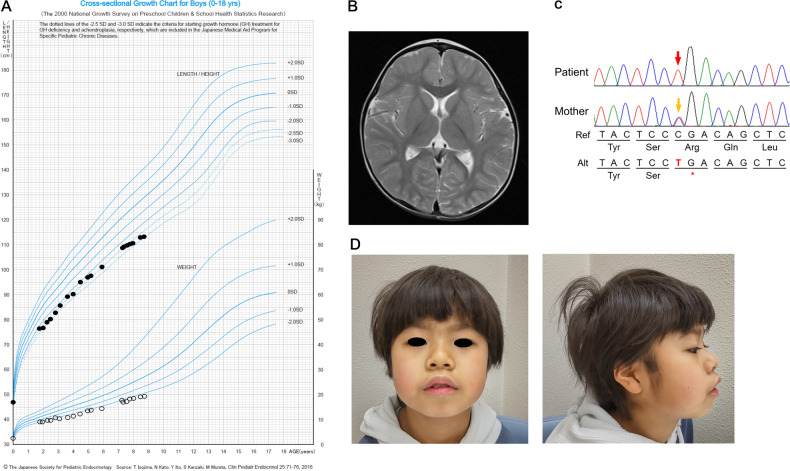
Clinical manifestations in this patient. **A** Growth curve. **B** Brain magnetic resonance imaging (T2-weighted image) of the patient at the age of 2 years. **C** Sanger sequencing results. A hemizygous c.736 C > T p.(Arg246*) mutation in the *KLHL15* gene was detected in the patient (red arrow), and his mother exhibited a heterozygous mutation (yellow arrow). A sample from the father was not available for genetic analysis. **D** Image of his present condition.

On admission at seven years and three months, he developed hypoglycemia (60 mg/dL) after 20 h of fasting. Plasma amino acid analysis (Supplementary Data 1), acylcarnitine analysis of dried blood spots, and urine organic acid analysis did not reveal any abnormalities. Moreover, no endocrine dysfunction, including thyroid or adrenal dysfunction, was observed. A growth hormone (GH) stimulation test with arginine (0.5 g/kg) and clonidine (0.15 mg/m^2^) did not reveal GH deficiency (Supplementary Data 2).

He is registered with the initiative on rare and undiagnosed diseases^10^, led by the Japan Agency for Medical Research and Development. Whole-exome sequencing was performed on the proband, and a hemizygous variant (c.736 C > T p.(Arg246*)) in *KLHL15* (NM_030624.3) was detected. Sanger sequencing confirmed that the hereditary origin of the variant was derived from the maternal lineage of the patient (Fig. 1C and Supplementary Data 3). This variant has not been registered in ClinVar (https://www.ncbi.nlm.nih.gov/clinvar/?gr=0), the general human genome databases of Tohoku Medical Megabank Organization (ToMMo)^11^, or the Genome Aggregation Database (gnomAD)^12^. This variant is considered a likely pathogenic variant according to the ACMG/AMP guidelines^13^ (PVS1_nonsense variant + PM2_not in gnomAD). Variants of disease genes related to hypoglycemia or periodic fever were not detected by whole-exome sequencing. He presented with attention-deficit/hyperactivity disorder (ADHD). An intelligence assessment using the Wechsler Intelligence Scale for Children-4th ed. (WISC-IV) at the age of 8 years and 11 months revealed a full-scale IQ of 47, a verbal comprehension index of 49, a perceptual reasoning index of 63, a working memory index of 54, and a processing speed index of 58. At the age of 10 years, he lived a stable life without medication, received rehabilitation, and participated in a social support program for developmental delays (Fig. 1D).

To date, six reported cases of male patients with variants in the *KLHL15* gene have demonstrated a clear association between specific genetic variants and corresponding phenotypes (Table 1 and Supplementary Data 4). XLID cases typically inherited recessively from carrier females, often result in intellectual disability in affected male offspring. In our case, the patient’s mother did not exhibit significant impairments in intelligence, and the patient did not present with facial abnormalities or abnormal endocrine function.

**Table 1:** Variants reported in *KLHL15*.

Variant no.	Nucleic acid	Amino acid	ClinVar	Polyphen-2 (Score)	Phenotype	References
1	g.24020361_24042839del	–	Pathogenic	NA	Psychomotor developmental delay, epilepsy, coarse facial features, anteverted nares, large mouth, short hands, abnormal genitalia with micropenis and bilateral cryptorchidism, the polymicrogyria-like appearance of the central areas and parietal lobes	Mignon-Ravix^5^
2	c.736 C > T	p.Arg246*	NR	NA	Moderate ID, frequent hypoglycemia, unbalanced diet	This study
3	c.1179del	p.Tyr394Ilefs*61	Pathogenic	NA	Mild to moderate ID, mild facial features	Hu^6^
4	c.1196dupA	p.Tyr399*	NR	NA	Motor developmental delay, speech delay	van der Ven^7^
5	c.1219 G > T	p.Glu407*	NR	NA	Developmental disorders	Fitzgerald^8^
6	c.1474 G > A	p.Val492Ile	Likely pathogenic	Probably damaging (0.996)	ID, facial asymmetry, CCA, cortical atrophy, seizures, hypergonadotropic hypogonadism, thyroid dysplasia	Karaca^2^
7	c.1596_1598del	p.Arg532del	NR	NA	Global developmental delay, coarse facial features, repetitive behavior, increased fatigability, poor feeding, gastroesophageal reflux	Caswell^9^

*CCA* cortical cerebellar atrophy, *ID* intellectual disability, *NA* not available, *NR* not registered.

Notably, previous reports have highlighted various clinical manifestations of XLID related to KLHL15. For instance, a patient with a large deletion of 22 kb, including exon 3 of KLHL15, exhibited severe intellectual disability, epilepsy, and anomalies in cortical development^5^. Other patients showed mild to moderate intellectual disabilities, mild facial abnormalities, motor development and speech delays, developmental disorders, and global developmental delay with additional features such as coarse facial features and abdominal endocrine dysfunction^2,6–9^.

Considering the available literature and our case, it is evident that XLID associated with KLHL15 involves intellectual disability, mild facial abnormalities, and potential hypogonadism. Additionally, the patient’s consumption of a small volume of food and lowered amino acid levels in the plasma suggest that an unbalanced diet may be a manifestation of this condition, contributing to episodes of hypoglycemia and short stature. Although the patient’s developmental levels have improved over time, they remain lower than those of peers. Further accumulation of clinical outcomes from other patients is necessary to gain a comprehensive understanding of the clinical course of this condition.

*KLHL15* is composed of four exons, including two noncoding exons and two coding exons^14^. The gene is expressed ubiquitously in human tissues, with particularly high expression observed in the adult brain, kidney, testis, and ovaries^14^. Within the adult brain, *KLHL15* exhibits the highest expression levels in regions such as the amygdala, cerebellum, corpus callosum, and thalamus^15^. This pattern of expression underscores the important role of *KLHL15* in maintaining brain function, and disruptions in this protein can contribute to intellectual disability and impairments in higher brain functioning, potentially manifesting as symptoms of ADHD, heightened fixation, and dietary imbalances. Additionally, although this particular patient did not exhibit endocrine function disorders, these disorders may also be associated with *KLHL15* defects.

In conclusion, we observed a nonsense variant (c.736 C > T p.(Arg246*)) in *KLHL15* in a male patient with X-linked intellectual disability (XLID). This variant causes a premature stop codon, resulting in a truncated transcript and functional disruption of *KLHL15*, leading to developmental disorders. Further research using mouse models or studying more patients is required to deepen our understanding of this disorder.

### Supplementary information

Supplementary Data 1

Supplementary Data 2

Supplementary Data 3

Supplementary Data 4

## Data Availability

The relevant data from this Data Report are hosted at the Human Genome Variation Database at 10.6084/m9.figshare.hgv.3308.
