# Supplementary material for: X-linked intellectual disability related to a novel variant of KLHL15
Source: Hum Genome Var. 2023 Jul 14;10:21. doi: 10.1038/s41439-023-00248-7 (PMC10349042; doi:10.1038/s41439-023-00248-7)
Supplement: Supplementary file 1 — Supplementary Data 1 [file 41439_2023_248_MOESM1_ESM.docx]

Supplementary Data 1. Plasma amino acids analysis

| Amino acid | Reference (μmol/L) | Age: 7 years 3 months |
| --- | --- | --- |
| Hydroxyproline | ≤ 21.6 | 13.1 |
| Threonine | 66.5-188.9 | 79.3 |
| Serine | 72.4-164.5 | 131.3 |
| Asparagine | 44.7-96.8 | 36.8 |
| Glutamic acid | 12.6-62.5 | 32.9 |
| Glutamine | 422.1-703.8 | 372.0 |
| Proline | 77.8-272.7 | 82.8 |
| Glycine | 151.0-351.0 | 153.4 |
| Alanine | 208.7-522.7 | 172.1 |
| Citrulline | 17.1-42.6 | 19.3 |
| Valine | 147.8-307.0 | 206.0 |
| Cystine | 13.7-28.3 | 9.9 |
| Methionine | 18.9-40.5 | 17.7 |
| Isoleucine | 43.0-112.8 | 61.2 |
| Leucine | 76.6-171.3 | 119.7 |
| Tyrosine | 40.4-90.3 | 47.7 |
| Phenylalanine | 42.6-75.7 | 45.6 |
| Histidine | 59.0－92.0 | 67.7 |
| Tryptophan | 37.0-74.9 | 39.9 |
| Ornithine | 31.3-104.7 | 41.1 |
| Lysine | 108.7-242.2 | 96.5 |
| Arginine | 53.6-133.6 | 45.6 |
| Total AA | 2068.2-3510.3 | 1943.8 |
| NEAA | 1381.6-2379.4 | 1210.2 |
| EAA | 660.0-1222.3 | 733.6 |
| BCAA | 265.8-579.1 | 386.9 |
| EAA/NEAA | 0.40-0.63 | 0.61 |
| BCAA/Total AA | 0.11-0.18 | 0.20 |
| Fisher ratio | 2.43-4.40 | 4.15 |

EAA: essential amino acids

BCAA: branched chain amino acids
