# Supplementary material for: X-linked intellectual disability related to a novel variant of KLHL15
Source: Hum Genome Var. 2023 Jul 14;10:21. doi: 10.1038/s41439-023-00248-7 (PMC10349042; doi:10.1038/s41439-023-00248-7)
Supplement: Supplementary file 2 — Supplementary Data 2 [file 41439_2023_248_MOESM2_ESM.docx]

Supplementary Data 2. The result of growth hormone (GH) stimulation.

A. Arginine (0.5g/kg) test

|  | GH (ng/mL) |
| --- | --- |
| Before | 7.31 |
| 30 minutes | 3.84 |
| 60 minutes | 2.93 |
| 90 minutes | 3.56 |
| 120 minutes | 0.97 |

B. Clonidine (0.15mg/m^2^) test

|  | GH (ng/mL) |
| --- | --- |
| Before | 1.32 |
| 30 minutes | 6.70 |
| 60 minutes | 8.92 |
| 90 minutes | 5.65 |
| 120 minutes | 1.86 |
