# Supplementary material for: X-linked intellectual disability related to a novel variant of KLHL15
Source: Hum Genome Var. 2023 Jul 14;10:21. doi: 10.1038/s41439-023-00248-7 (PMC10349042; doi:10.1038/s41439-023-00248-7)
Supplement: Supplementary file 3 — Supplementary Data 3 [file 41439_2023_248_MOESM3_ESM.docx]

Supplementary Data 3. Sanger sequence method for *KLH15* in this case

*KLHL15* variants in the PCR products were confirmed using Sanger sequencing with an ABI PRISM 3500xl autosequencer (Life Technologies, Carlsbad, CA, USA), using genomic DNA from the patient and his mother as a PCR template. Genomic DNA (gDNA) was extracted from peripheral blood leukocytes using QuickGene-610 L (Fujifilm, Tokyo, Japan), according to the manufacturer’s protocol. The PCR mixture was prepared in a 10-μL volume contained 1.0 μL of 10× Ex Taq Buffer (Takara, Otsu, Shiga, Japan), 0.8 μL of 2.5 mM dNTP Mixture (Takara), 0.05 μL of Ex Taq HS (Takara), 10 μM forward and reverse primers, and 10 ng gDNA. PCR conditions were as follows: denaturing at 94°C for 2 min; 5 cycles at 94°C for 30 s and 68°C for 30 s; 5 cycles at 94°C for 30 s, 65°C for 30 s, and 72°C for 30 s; 5 cycles at 94°C for 30 s, 63°C for 30 s, and 72°C for 30 s; 25 cycles at 94°C for 30 s, 58°C for 30 s, and 72°C for 30 s; and extension at 72°C for 3 min.

Forward primer; AGAAAGTAGGTGATCTTAGCAGGA

Reverse primer; CGGATGCGGCTTGACTTCAT
