# Supplementary material for: X-linked intellectual disability related to a novel variant of KLHL15
Source: Hum Genome Var. 2023 Jul 14;10:21. doi: 10.1038/s41439-023-00248-7 (PMC10349042; doi:10.1038/s41439-023-00248-7)
Supplement: Supplementary file 4 — Supplementary Data 4 [file 41439_2023_248_MOESM4_ESM.pptx]

## Slide 1
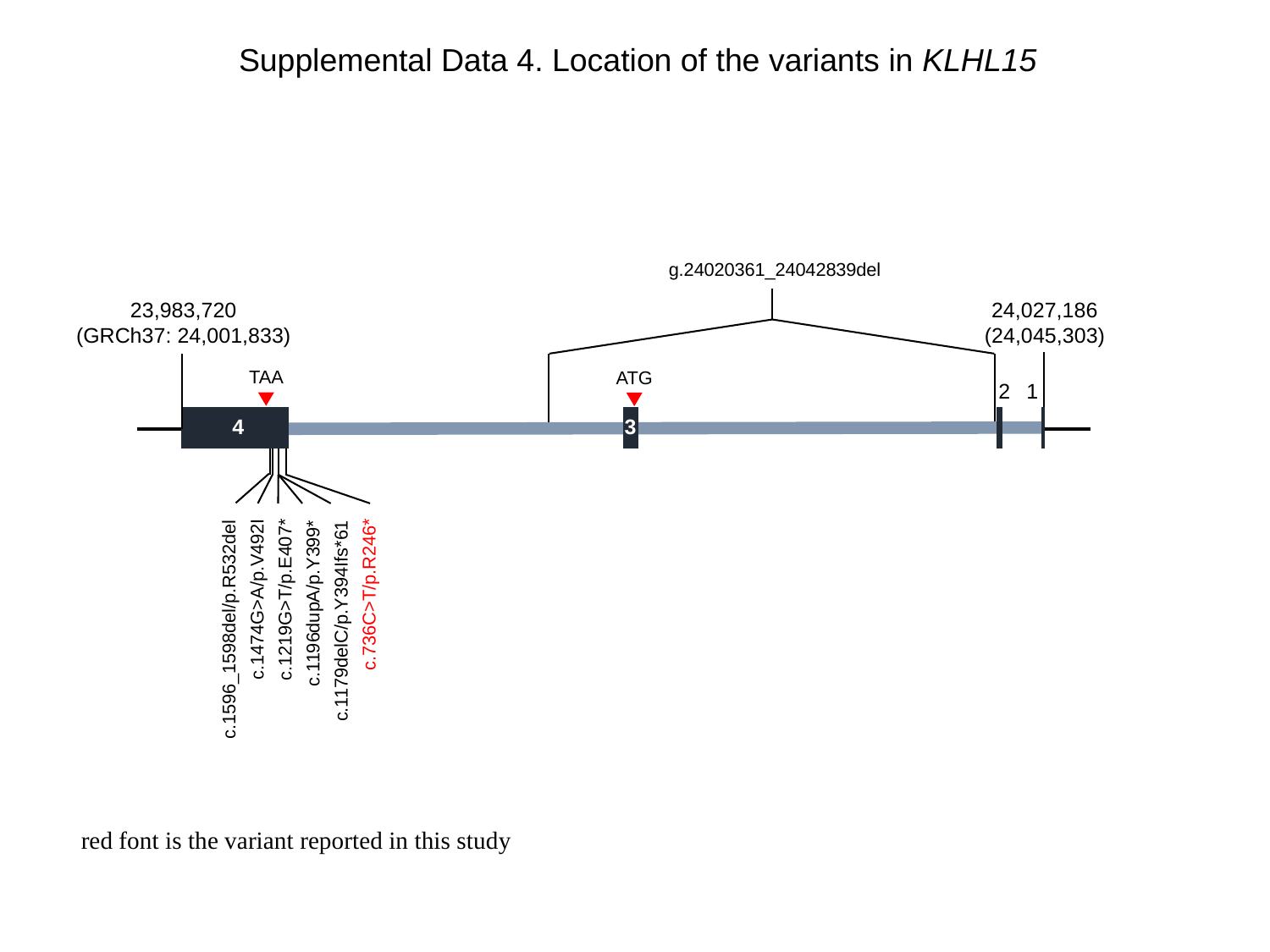

Supplemental Data 4. Location of the variants in KLHL15
g.24020361_24042839del
23,983,720
(GRCh37: 24,001,833)
24,027,186
(24,045,303)
TAA
ATG
2
1
4
3
c.736C>T/p.R246*
c.1474G>A/p.V492I
c.1219G>T/p.E407*
c.1196dupA/p.Y399*
c.1179delC/p.Y394Ifs*61
c.1596_1598del/p.R532del
red font is the variant reported in this study
